# Normalization of *NPM1* mutant transcript to the wild‐type transcript

**DOI:** 10.1002/jha2.579

**Published:** 2022-09-27

**Authors:** Lawrence J. Jennings

**Affiliations:** ^1^ Department of Pathology Feinberg School of Medicine Northwestern University Chicago Illinois USA

**Keywords:** MRD, Measurable Residual Disease, AML, *NPM1*

## Abstract

Current guidelines recommend that Acute Myeloid Leukemia (AML) patients with *NPM1* mutations should be monitored for measurable residual disease by quantifying the transcripts and normalizing them to *ABL1* transcripts. In this short report, a simple and highly accurate method to quantify the *NPM1* mutant transcript normalized to the wild‐type *NPM1* transcript is presented. The percent mutant transcript correlates very well to the corresponding mutant allele frequency as determined by DNA‐based methods allowing direct comparison of investigational studies that use RNA‐based or DNA‐based methods for monitoring *NPM1* mutations.

## 1 INTRODUCTION

The European LeukemiaNet (ELN) published a consensus document and later an update to that document that included recommendations for monitoring minimal/measurable residual disease in *NPM1*‐mutated AML patients [1, 2]. For *NPM1*‐mutated patients, ELN recommends molecular MRD assessment every 3 months for 24 months after the end of treatment in the bone marrow and peripheral blood, or every 4–6 weeks in peripheral blood. Furthermore, “for reasons of sensitivity for quantitative polymerase chain reaction (qPCR)”, ELN recommends the use of cDNA over DNA for genes that are well expressed in AML cells, which includes *NPM1*. However, the consensus document also recommends reporting the *NPM1* cDNA copy number and Ct value as compared to the housekeeping gene, *ABL1*. The reasons for normalizing to *ABL1* are not stated but are likely historical.

Reporting *NPM1* residual disease burden relative to *ABL1* expression or as log change, for that matter, creates a challenge in interpretation for clinicians and standardization for laboratories. Similar challenges with BCR‐ABL1 transcript quantification plagued both clinicians and laboratories for years as attempts were made to normalize methods and housekeeping genes across clinical laboratories [3]. Alternatively, one can determine *NPM1* residual disease burden by using DNA and assessing variant allele frequency. Investigators compared RNA‐based (RT‐qPCR) and DNA‐based (droplet digital PCR [ddPCR] and Next‐Generation Sequencing [NGS]) methods for measuring residual disease in *NPM1*‐mutated AML [4]. For this study, *NPM1* cDNA was also normalized to *ABL1* cDNA. Not surprisingly given the lability of RNA, they noted that *NPM1* transcripts as compared to variant allele frequency as a surrogate for leukemic cells “fluctuated considerably between different follow‐up samples”.

For purposes of monitoring minimal/measurable residual disease, *NPM1* is treated like a fusion product that must be normalized to a housekeeping gene. However, mutated *NPM1* alleles have a corresponding normal allele with the same promoter and are therefore likely to have similar levels of expression. Herein, it is shown that 1) *NPM1* is expressed at a far higher rate than *ABL1* and therefore should not be normalized to that housekeeping gene, and 2) quantifying mutated *NPM1* transcript as compared to the wildtype *NPM1* transcript correlates very well to the variant allele frequency as determined by DNA‐based methods.

## 2 METHODS

### 2.1 Samples and extraction

Samples were collected for clinical testing. DNA was extracted using QIAamp DNA Blood Mini Kit (Qiagen, Germantown, Maryland) following the manufacturer's instructions. RNA was extracted using NucleoSpin RNA Blood Midi Kit (Takara Bio Inc, Shiga, Japan), following the manufacturer's instructions. Nucleic acid was quantified using NanoDrop Lite Plus Spectrophotometer (Thermo Fisher Scientific Inc.)

### 2.2 ddPCR and one‐step RT‐ddPCR

To quantify the mutant allele when allele frequency was below 1%, ddPCR was performed using ddPCR Supermix for Probes from Bio‐Rad Laboratories (Cat# 1863027; Hercules, CA). To quantify transcripts, one‐step droplet digital RT‐PCR was performed using a one‐step RT‐ddPCR Advanced Kit for Probes from Bio‐Rad Laboratories (Cat# 1864022; Hercules, CA). The sequences of primers and probes together with their reaction concentrations are given in Table S1. The *NPM1* probes are all minor groove binder probes to improve specificity (Thermo Fisher Scientific Inc.). To assess the *NPM1* transcript as compared to the *ABL1* transcript, the *NPM1* wild‐type probe was combined with the *ABL1* probe. To assess *NPM1* mutant transcript as compared to *NPM1* wild‐type transcript, probes to mutant types A, B, and D were combined with *NPM1* wild‐type probe. Because of the high level of the transcript, the RNA input was limited from 4 to 100ng per well. The input of 4ng allowed quantification of the *NPM1* wild‐type transcript and higher inputs of 20 or 100 ng allowed detection of rare *NPM1* mutant transcripts. Thermal‐cycling conditions were as follows: 95°C × 10 min (1 cycle), 94°C × 30 s (ramp rate 2°C/s), 55°C × 60 s (ramp rate 2°C/s) (40 cycles), 98°C × 10 min (1 cycle), and a 12°C hold. The one‐step RT‐ddPCR also had an additional reverse transcription step at the start (50°C × 60 min). After cycling, the 96‐well PCR plate was loaded on Bio‐Rad's QX200 droplet reader, which reads the droplets from each well of the plate. Analysis of the data was performed with QuantaSoft Analysis Pro 1.0.596.

## 3 RESULTS

**FIGURE 1:**
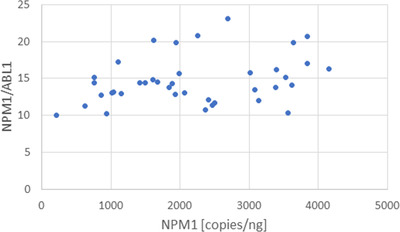
Ratio of *NPM1* to *ABL1*. Clinical samples were collected for various indications and total RNA was extracted within 2 h–3 days. Quantification of copies per nanogram shows a consistent ratio of *NPM1* mRNA copies to *ABL1* mRNA copies across a wide range of amplifiable RNA (average, 14.7; standard deviation, 3.2)

To compare *NPM1* expression to *ABL1* expression, a series of 45 clinical samples known to be negative for *NPM1* mutations and *ABL1* rearrangements were diluted and tested by one‐step ddRT‐PCR. Despite diluting up to 200‐fold, some samples were outside the analytical measurement range leaving 39 samples with useful data. As shown in Figure 1, The average expression of *NPM1* to *ABL1* was approximately 15‐fold greater across a wide range of samples of varying yields (average = 14.97, standard deviation = 3.2).

**FIGURE 2:**
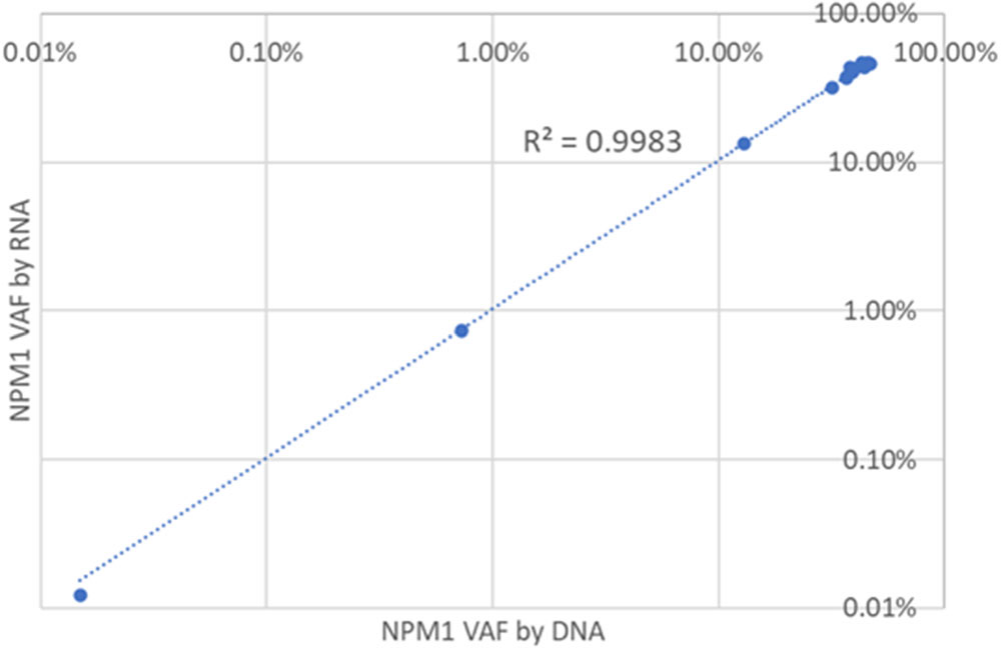
Variant allele frequency from RNA versus DNA. Clinical samples were collected with known *NPM1* mutations. Variant allele frequency was quantified from DNA using either NGS or droplet digital polymerase chain reaction (ddPCR). Variant allele frequency was also quantified from total RNA using ddRT‐PCR. The variant allele frequencies were highly correlated (*R* ^2^ = 0.9983)

To quantify the *NPM1* mutant allele, a multiplex probe mix of mutant types A, B, and D, all labeled with FAM, were combined with the *NPM1* wildtype probe (labeled VIC). A dilution series demonstrated single‐well positivity and linearity to 0.0005% (Figure S1). A series of 20 negative samples showed no false positive droplets, indicating that the lower limit of detection is only limited by the amount of amplifiable RNA that is available (data not shown). Next, 10 diagnostic and follow‐up samples that had been previously identified through NGS as *NPM1*‐positive were sequentially selected. For samples with a mutant allele burden below 1%, *NPM1* mutant allele was quantified with ddPCR. All positive samples were also tested for *NPM1* mutant versus wild‐type expression. As shown in Figure 2, the variant allele frequencies determined by normalizing to the wildtype transcript were highly correlated to the variant allele frequency determined by NGS or ddPCR (*R* ^2^ = 0.9983).

## 4 DISCUSSION

The expression of *NPM1* is approximately 15‐fold higher than that of *ABL1* with a standard deviation of 3.2. Expression was determined using one‐step RT‐ddPCR to eliminate bias due to complementary DNA transcription or amplification efficiency, and this estimate should therefore be quite accurate. However, for these studies, the amplicon size of *NPM1* is longer than *ABL1* (169 bp and 124 bp, respectively), and the ratio might therefore even be higher. The specific *ABL1* primers and probes were chosen because these are often used for the normalization of *NPM1* mutation burden [5], and the intent was to highlight the high expressivity of *NPM1* as compared to the amplicon typically used for normalization.

When the *NPM1* mutant transcript is normalized to the *NPM1* wild‐type transcript, the percent *NPM1* mutation correlates very well to the corresponding variant allele frequency as determined by DNA testing. This is not surprising given that the mutant and normal *NPM1* alleles have the same promoter and therefore are expected to have the same level of expression. Moreover, the stability and integrity of the mutant and wild‐type transcript should be the same ensuring accurate quantification even in compromised samples.

In this short report, a simple and highly accurate method to quantify the *NPM1* mutant transcript level was presented. This method could certainly be widely adopted across other clinical laboratories to better stratify patients for therapies and clinical trials. However, that is not required. Other laboratories may also keep their current approach of qPCR or RNA sequencing and simply normalize to the wild‐type transcript level. Standardization of methods for *NPM1* mutant transcript quantification would not be necessary providing a stated lower limit of detection could be achieved. Furthermore, because mutant transcript normalized to the wild‐type transcript is highly correlated with DNA mutant allele frequency, investigational studies could reasonably target either biomarker and meta‐analyses can be performed. However, the increased sensitivity of RNA testing does make *NPM1* mutant transcript detection with normalization to the wild‐type transcript the ideal approach, especially for peripheral blood samples where the sensitivity may be 5‐ to 10‐fold lower than that of bone marrow [6, 7].

## CONFLICT OF INTEREST

The author declares no conflict of interest.

## FUNDING INFORMATION

All funding was sourced through intradepartmental development grants.

## ETHICS STATEMENT

Verbal consent to diagnostic testing was provided by all participants.

## Supporting information

Figure S1 Serial dilution of control sample with known *NPM1* mutation. Detection by ddRT‐PCR shows linearity to 0.0005% (*R* ^2^ = 0.9987).

Supplemental Table 1: Primers and probes.
