# Supplementary figures and images for: Normalization of NPM1 mutant transcript to the wild‐type transcript
Source: EJHaem. 2022 Sep 27;3(4):1343–5. doi: 10.1002/jha2.579 (PMC9713034; doi:10.1002/jha2.579)

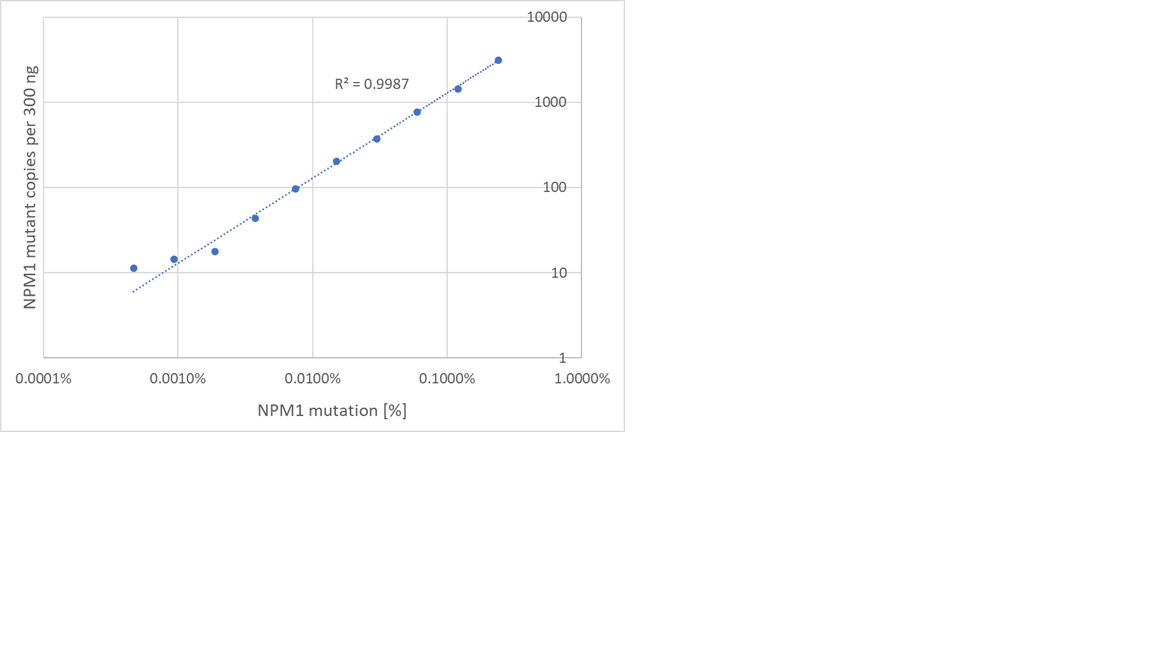

Supplement: Supplementary file 1 — Figure S1 Serial dilution of control sample with known NPM1 mutation. Detection by ddRT‐PCR shows linearity to 0.0005% (R 2 = 0.9987). [file JHA2-3-1343-s002.tif]

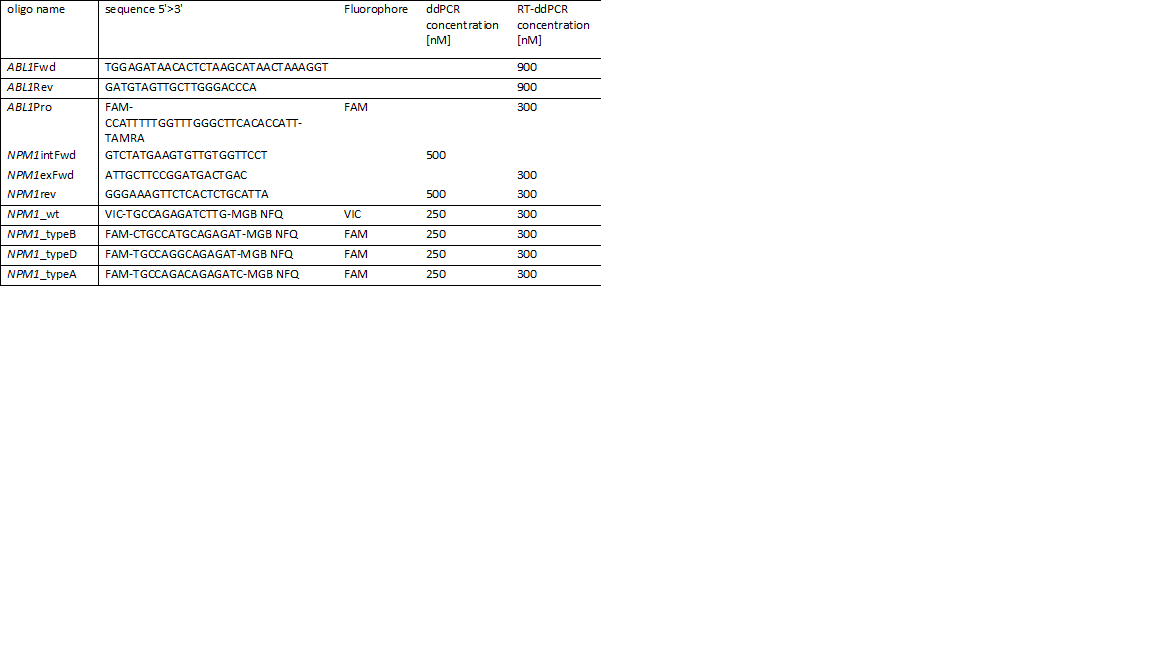

Supplement: Supplementary file 2 — Supplemental Table 1: Primers and probes. [file JHA2-3-1343-s001.tif]
